# Developmental Differences in Holistic Interference of Facial Part Recognition

**DOI:** 10.1371/journal.pone.0077504

**Published:** 2013-10-31

**Authors:** Kazuyo Nakabayashi, Chang Hong Liu

**Affiliations:** 1 Department of Psychology, The University of Hull, Hull, United Kingdom; 2 School of Design, Engineering, and Computing, Bournemouth University, Dorset, United Kingdom; Federal University of Rio de Janeiro, Brazil

## Abstract

Research has shown that adults’ recognition of a facial part can be disrupted if the part is learnt without a face context but tested in a whole face. This has been interpreted as the holistic interference effect. The present study investigated whether children of 6- and 9–10-year-olds would show a similar effect. Participants were asked to judge whether a probe part was the same as or different from a test part whereby the part was presented either in isolation or in a whole face. The results showed that while all the groups were susceptible to a holistic interference, the youngest group was most severely affected. Contrary to the view that piecemeal processing precedes holistic processing in the cognitive development, our findings demonstrate that holistic processing is already present at 6 years of age. It is the ability to inhibit the influence of holistic information on piecemeal processing that seems to require a longer period of development into at an older and adult age.

## Introduction

Leder and Carbon [11] demonstrate that when a facial part such as eyes is learnt in isolation, the recognition of this part can be disrupted if it is presented in a whole face at test. The result shows that adult observers are unable to ignore irrelevant parts in the whole face. The authors argue that this interference is an essence of holistic face processing. Here, we investigate whether 6- and 9–10-year-old children would exhibit the same holistic interference effect.

One of the key questions in face recognition has been whether there is a qualitative difference in the way children and adults process faces. A classic hypothesis, *encoding switch hypothesis*, states that children recognise faces in a piecemeal fashion, with a focus on constituent facial parts during early years of life, but at around 8 years of age their processing starts to shift to a holistic strategy [4]. More recently, an increasing number of studies have shown that children process faces as a perceptual whole just like adults do. That is there is no qualitative difference in the way children and adults use holistic information to perceive, store, and recognise faces [6], [12], [17], [18], [24].

Using a part-whole paradigm, Tanaka and Sengco [25] examined college students’ memory for a facial part (i.e., eyes, nose, and mouth). Participants first learnt a number of target faces with a name attached to each face, and then in a subsequent recognition test they were presented with a verbal statement of one of the target faces (e.g., Bob’s nose), followed by the presentation of two pictures. The task was to indicate which one of the pictures depicted the target feature (e.g., Bob’s nose). Performance was tested in part (i.e., a part in isolation), original intact face, and new configuration (i.e., eyes were moved closer together or further apart from each other) conditions. The results showed that the recognition of a facial part by the college students was better when tested in the original intact face, worse when tested in the face with new configuration, and worst when tested in isolation. These findings were attributed to holistic processing in face recognition in which facial features and their spatial layout are processed as a perceptual whole, rather than constituent parts.

Tanaka et al., [24] extended the paradigm to children of 6-, 7-, and 8-year-olds. The children were asked to learn 4 face-name pairs. In the subsequent recognition test, they were shown target-part and probe-part in a part condition and target-face and probe-face in a whole condition, and identified which one is the correct target feature (“Which one is Tom’s nose?”). The authors found that all children recognised parts better when the parts were tested in a whole face than in isolation. This has been taken as evidence of a holistic representation of a face by 6 years old. It shows that children’s face processing does not change from a part-based to holistic strategy from this age. Subsequent studies reported evidence of holistic encoding even among 4- and 5-year-olds whose part recognition was better in a whole than part condition [17]. Pellicano, Rhodes, and Peters [18] further reported no qualitative difference in face processing between adults and 4-year-olds. Similarly, Seitz [19] also found no qualitative change in the development of visual processing of faces as children get older, but their recognition performance becomes more accurate with age. However, there is also evidence for the encoding switch hypothesis. Hay and Cox [9] found better whole face recognition by their 9- and 10-year-olds and better part (i.e., eyes) recognition by 6- and 7-year-olds.

None of the developmental studies have examined whether part recognition following *part learning* would also be affected by the presentation of a whole face at test. These studies investigated part recognition following whole learning. However, as Leder and Carborn [11] suggest, it is important to address whether the whole advantage also arises following part learning. The idea behind this is that if part learning results in a part representation, then showing the part in a whole face or in isolation at test should have little effect on the recognition of the part. In their study, adult participants learnt 6 face-name pairs either in a part or whole condition. In the following recognition test, the participants were presented either with the 6 names and one of the studied faces in a whole condition or the names and one of the studied parts in a part condition. The task was to select an appropriate name of the target part/face. The results showed that wholes were recognised better only following whole learning. When parts were learned, presenting a whole face at test impaired the recognition of the parts. These results indicate that it is very difficult to ignore irrelevant information available in a whole face.

It is apparent from the Leder and Carbon [11] study that the interaction between a part and whole plays a key role in adult face recognition. However, it remains uncertain as to whether children’s memory of facial parts are equally affected and impaired by wholes as a result of the same holistic processing because the current knowledge about the development of holistic processing is limited to the manipulation where a whole face is learned. Moreover, surprisingly little attention has been paid to address the robustness of part processing among children, relative to that of adults, even though face feature encoding is known to play a key role in face processing [23], [25]. The current study, therefore, investigated these neglected issues. The aims were to find out 1) whether children’s part recognition following part learning would also be influenced by the presentation of a whole face at test, and 2) how robust children’s part processing might be and how this could be developed as children get older. Following Leder and Carborn [11], we used eyes as part stimuli.

In order to address these questions, we took a different approach to previous developmental studies by examining the effects of *holistic interference* on part recognition. We examine whether there would be a developmental shift in the effects of holistic interference on part recognition. If children rely predominantly on a part representation, then they should show either weak or no holistic interference when a whole face is presented at the time of encoding, recognition, or both. Alternatively, if part processing becomes less dominant with age, then adults would show stronger holistic interference than children.

We conducted a systematic examination into the effects of context on encoding and recognition processes, and the effects of probe (part/whole) – test (part/whole) context congruency on part recognition. This resulted in four probe-test conditions: 1) part-part; 2 part-whole; 3) whole-part; 4) whole-whole conditions. In the part-part condition, participants saw eyes in isolation as a probe, and tested with eyes in isolation, hence this condition examined part recognition following part learning. In the part-whole condition, part learning was followed by whole test whereby test eyes were presented in a whole face. In the whole-part condition, whole learning was followed by part test. Thus, the part-whole and whole-part conditions examined whether a time when a whole face is presented, either during encoding or retrieval, would make a difference to part recognition performance. In the whole-whole condition, whole learning was followed by whole test using a composite face or the original face. During ‘same-part’ trials probe eyes were shown in the original face, but were tested in the context of another face. Therefore, the two sets of eyes were of the same face, but the context in which they were presented differed. During ‘different-part’ trials probe eyes were shown in the original face, but a different pair of eyes were placed and tested in the original face. Thus, the two sets of eyes were of two different faces, but the context in which they were placed was identical. We used this condition to examine the ability to extract a part from a face without being influenced by irrelevant facial context. In all four conditions, participants were asked to judge whether probe and test eyes were the same or different.

The following predictions were made. Part recognition should be best in the part-part condition for all age groups, as it creates no interference. If holistic processing becomes more developed with age, it would be more difficult for adults to ignore irrelevant facial information in a whole face than two groups of children. Hence, adults would show worst performance in the whole-whole condition. However, if age does not affect the processing of part information, then all the groups’ performance would be disrupted similarly by the presentation of a whole face.

## Materials and Methods

### Ethics Statement

The study was approved by the Research Ethics Committee of the Department of Psychology, The University of Hull and Lancaster University. All adult participants gave written informed consent while parents gave written informed consent on behalf of their children. All the participants were treated in accordance with the principles expressed in the Declaration of Helskinki.

### Participants

Three White British groups were recruited for this study: thirty-six 6-year-olds (*M* = 6 years and 2 months; 17 boys and 19 girls); thirty-six 9- to 10-year olds (*M* = 9 years and 9 months; 18 boys and 18 girls); and forty-eight adults (*M* = 21 years; 22 males and 26 females). We chose these age categories because prior studies that examined developmental changes in facial part processing had employed similar age groups [9], [16]. The children were recruited from two primary schools in West Yorkshire while the adult participants were undergraduate students in the psychology department at University of Hull. All the participants had normal or corrected-to-normal vision.

### Stimuli and Apparatuses

**Figure 1:**
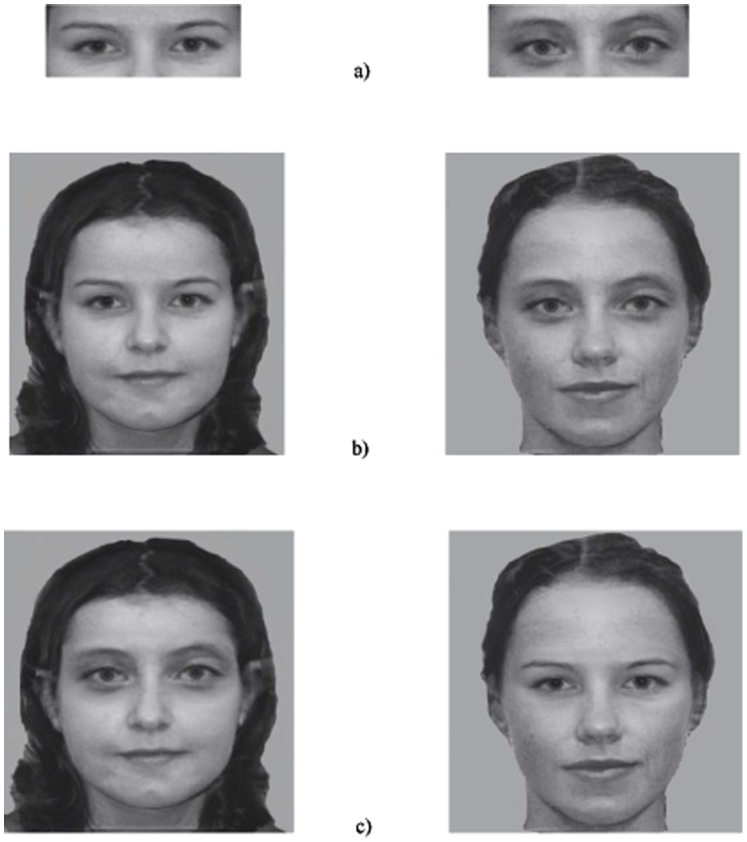
Example images: a) isolated eyes; b) original intact faces; c) composite faces, with the eyes placed in another face. Images in this figure are used for illustrative purposes only. They are not the original stimuli, but are morphed images to protect the identity of the children.

Stimuli were created from 80 greyscale images of White British children composed of 40 boys and 40 girls, aged between 6 to 10 years old (see Figure 1 for an illustration). The facial images were taken from a database at the psychology department in the University of Lancaster. All the faces, which were unfamiliar to participants, were in a frontal view with neutral expressions showing no teeth. The background was edited out using Adobe Photoshop 5.5 and was replaced with a neutral grey background. The boys’ and girls’ faces were used equally frequently across conditions and no face was used more than once across conditions, resulting in two stimulus sets. Each stimulus set consisted of 40 probe - test pairs of images, with each containing 10 probe part - test part pairs, 10 probe part - test whole pairs, 10 probe whole - test part pairs, and 10 probe whole - test whole pairs.

‘Part’ stimuli were created by extracting the eye region (the eyebrows and eyes) using The Home Gene Splicing Kit (You Betcha Software), allowing the extraction of this region without causing any changes to the remaining face parts. Composite whole face stimuli (those to be used in the whole-whole condition) were created by using Graphicconventer (You Betcha Software) with the following steps. Firstly, faces with clear eyes and eye regions were selected (i.e., eyes not covered by fringe and no shadows around the eye region). Secondly, of those selected faces, faces that were similar in skin colour, age, gender, and head pose were paired. Finally, the eye region of one child was extracted and pasted onto the same region of another child, without causing any additional changes to the remaining parts of the face.

All the part images were reduced to a standardised size of 3.5 cm×1.3 cm (3.3°×1.2°) and all the whole images were standardised to the size of 5.8 cm×5.4 cm (5.5°×5.2°), using Adobe Photoshop 5.5. The images were presented using SuperLab Pro, and were viewed from a distance of 60 cm.

### Design and Procedure

A mixed factorial design was used, with age (6-/9–10-year-olds/adults) as the between-participants factor, and probe (isolated eyes/whole face) and test (isolated eyes/whole face) as within-participant factors. Participants engaged in a same-different recognition task whereby they indicated whether probe eyes and test eyes were of the same child or two different children. Practice trials were run in order to ensure that each child understood the instructions. The children were tested in a quiet room in the school while the adults were tested in a lab room in the university. Each participant was tested in all four probe - test conditions. There were 10 trials per condition, with an equal number of ‘same-’ and ‘different-part’ trials. The order of trials was randomised within and across participants. In addition, the order of condition was counterbalanced across participants.

At the beginning of the experiment each child was given an explanation about the nature of the stimuli, and was provided with the following instructions: ‘We are going to play a game. You will see a face/pair of eyes that you need to look at carefully, and then you will see another face/pair of eyes. What you have to do is to decide whether the eyes shown first are the same as or different from the eyes shown second. The eyes in the two photos are sometimes the same but they are sometimes different’.

Each trial began with a 5 second probe image, followed by a 5 second ISI, then a 5 second test image. This was followed by the question “Are the eyes shown first the same as or different from the eyes shown second?” The participant was then told to provide a ‘same’ or ‘different’ response by pressing one of two designated keys on the keyboard. It took approximately 25 minutes for the 6-year-olds and 20 minutes for the 9–10-year-olds and adults to complete the experiment, with short breaks between the conditions.

## Results

Recognition accuracy was examined using the signal detection theory [7], [8], [21], [22]. As in other studies [3], [13], [14] we calculated sensitivity (*A′*) and response bias (*B′′D*) following Donaldson [7]. Table 1 shows means and standard deviations for *A′*, *B′′D*, proportions of hits and false alarms (FAs) in each of the four conditions. For *A′*, a value of 0.5 indicates chance performance, and a value of 1 indicates perfect performance. For *B′′D,* values above 0 indicate a conservative bias and values 0 below a liberal bias. We conducted a 4 (condition: part-part, part-whole, whole-part, whole-whole)×3 (group: 6-, 9–10-year-olds, adults) repeated measures Analysis of Variance (ANOVA), with condition as the within-participant factor and group as the between-participants factor for *A′*, *B′′D,* hit and FA proportions.

**Table 1:** Means and standard deviations for *A′* (sensitivity), proportions of hits and false alarms (FA), and *B′′D* (bias) as a function of condition and group.

	Probe-Test		Part-Part	Part-Whole	Whole-Part		Whole-Whole	
		Group	M (SD)	M (SD)		M (SD)		M (SD)
*A′*		6 years	.90 (.11)	.78 (.15)		.80 (.13)		.63 (.35)
		9–10 years	.94 (.09)	.83 (.18)		.88 (.10)		.87 (.10)
		Adults	.88 (.12)	.89 (.09)		.88 (.10)		.81 (.10)
Hits		6 years	.82 (.20)	.61 (.27)		.62 (.24)		.53 (.28)
		9–10 years	.84 (.10)	.66 (.27)		.72 (.20)		.71 (.23)
		Adults	.81 (.14)	.82 (.16)		.84 (.14)		.66 (.23)
FAs		6 years	.11 (.12)	.19 (.20)		.17 (.19)		.25 (.21)
		9–10 years	.16 (.15)	.11 (.14)		.10 (.15)		.11 (.14)
		Adults	.25 (.14)	.16 (.16)		.19 (.20)		.20 (.17)
*B′′D*		6 years	−.02 (.14)	−.03 (.21)		0 (.18)		.04 (.40)
		9–10 years	0 (.11)	0 (.15)		.06 (.17)		.03 (.16)
		Adults	−. 09 (.11)	−.02 (.13)		−.03 (.14)		−.04 (.14)

*Note:* For the *A* prime measure large values indicate a greater ability to discriminate between probe and test items. For *B′′D,* values above 0 indicate a conservative bias and values 0 below indicate a liberal bias.

**Figure 2:**
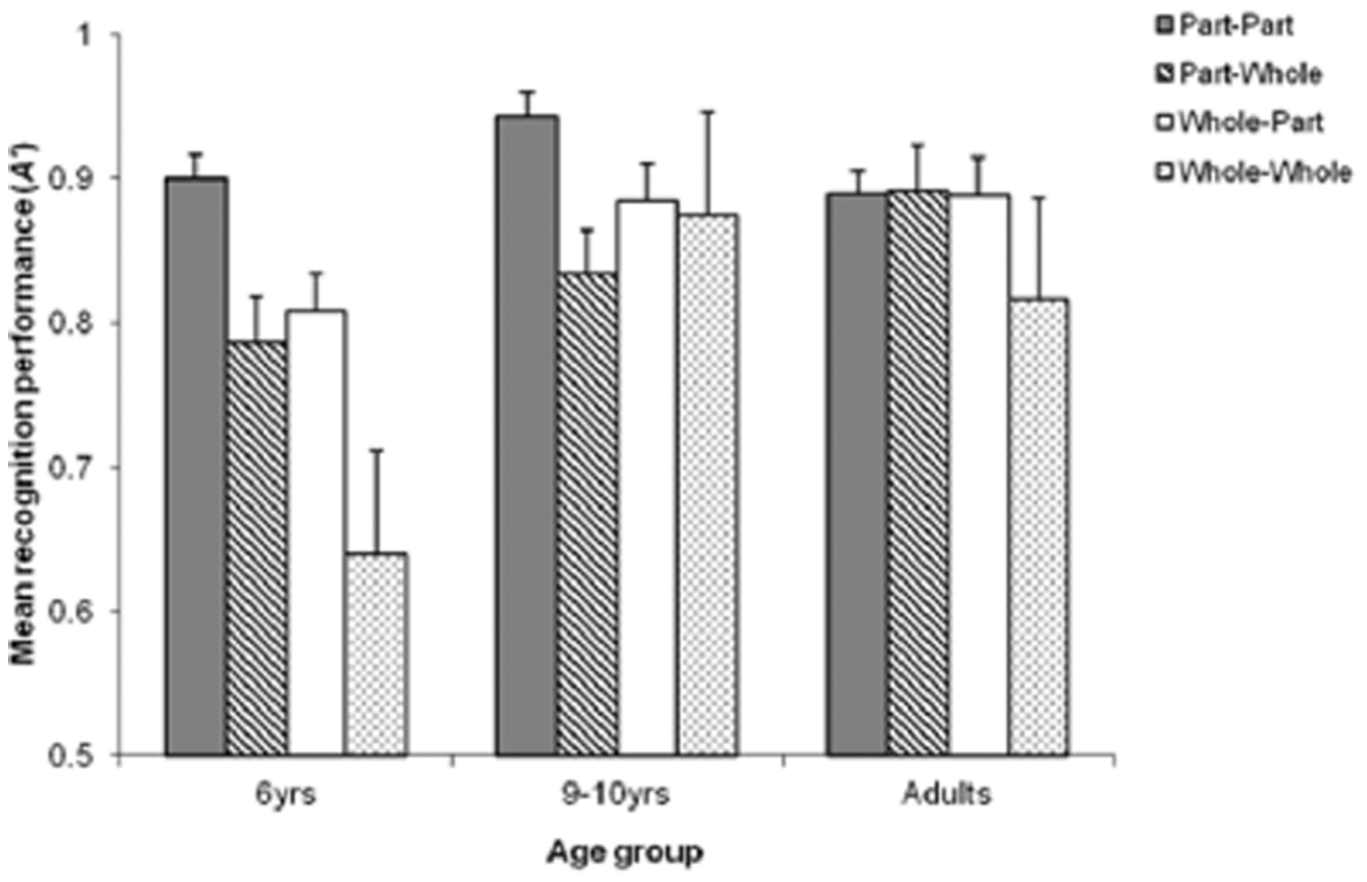
Recognition performance (*A′*) as a function of condition and age group. Error bars represent standard error.

For *A′*, results of the analysis showed effects of condition *F*(3,345) = 17.68, *p*<.001 and group *F*(2,115) = 13.26, *p*<.001. However, these results were qualified by the two-way interaction *F*(6,345) = 5.41, *p*<.001 (see Figure 2). We conducted a separate simple main effects analysis for each condition, which revealed that the interaction was due to a lack of group difference in the part-part condition F(2,115) = 2.52, *p*>.05, but clear differences in the other conditions. In the part-whole condition *F*(2,115) = 5.27, *p*<.01, the adults performed better than the 6-year-olds. In the whole-part condition *F*(2,115) = 5.64, *p*>.01, the 9–10-year-olds and adults performed better than the 6-year-olds, with no difference between the two older groups (*p*>.05). The same pattern of findings was found in the whole-whole condition *F*(2,115) = 11.79, *p*<.001, with the two older groups performing better than the 6-year-olds. These results indicate that group differences emerged only when a whole face was presented, either once or twice, with the 9–10-year-olds and adults performing better than the 6-year-olds under these conditions.

We conducted an additional one-way ANOVA for each group to examine the effect of face context on part recognition, which showed a significant main effect of condition for all the groups. The 6-year-olds performed best in the part-part condition, followed by the whole-part and part-whole conditions, and the worst performance in the whole-whole condition *F*(3,105) = 10.54, *p*<.001. The 9–10-year-olds performed better in the part-part condition than either part-whole or whole-whole condition *F*(3,105) = 4.74 *p*<.01. The adults showed poorer performance in the whole-whole than any of the other three conditions *F*(3,135) = 7.11, *p*<.001. For all the groups, the results of part-whole and whole-part conditions were comparable (*p*>.05).

For *B′′D*, results of the analysis showed an effect of group *F*(2,115) = 6.50, *p*<.01, with the adults (*M* = −.048) showing more liberal responding than the 6- or 9–10-year-olds (*Ms* = −.01 and.03, respectively). Neither condition *F*(3,345) = 1.93, *p*>.05 nor the two-way interaction *F* <1 was significant.

For hit proportions, results of the analysis showed effects of condition *F*(3,345) = 18.85, *p*<.001, group *F*(1,115) = 11.50, *p*<.001, and the two-way interaction *F*(6,345) = 4.74, *p*<.001. Simple main effects analyses showed that the interaction was due to a lack of group difference in the part-part condition, but clear differences in the other conditions. In the part-whole *F*(2,115) = 8.98, *p*<.001 and whole-part conditions *F*(2,115) = 13.12, *p*<.001, the adults produced more hits than the two younger groups. In the whole-whole condition *F*(2,115) = 4.73, *p*<.05, the 9–10-year-olds produced more hits than the 6-year-olds.

As with the *A′* data, we also performed a separate analysis for each group, which showed a significant main effect of condition for all the groups. The 6-year-olds produced more hits in the part-part condition, followed by the whole-part and part-whole conditions, and worst in the whole-whole condition *F*(3,105) = 11.93, *p*<.001. The 9–10-year-olds produced more hits in the part-part condition, followed by the part-whole and whole-whole conditions *F*(3,105) = 4.65, *p*<.01. The adults produced more hits in the part-part than whole-whole condition *F*(3,105) = 11.37, *p*<.001. Consistent with the *A′* results, group differences in the hit rates were found in all conditions except the part-part condition. When a whole face was presented, either the adults or 9–10-year-olds produced more hits than the 6-year-olds.

For FA proportions, results of the analysis showed effects of group *F*(1,115) = 265.97, *p*<.001 and the two-way interaction *F*(6,345) = 3.86, *p*<.01. A main effect of condition was non-significant *F*(3,345) = 1.41, *p*>.05. Simple main effects analyses showed that the interaction was due to group differences in the part-part *F*(2,115) = 10.26, *p*<.001 and whole-whole conditions *F*(2,115) = 5.63, *p*<.01, but absence of group differences in the part-whole *F*(2,115) = 2.14, *p*>.05 or whole-part condition *F*(2,115) = 2.71, *p*>.05. In the part-part condition, the adults produced more FAs than the two younger groups. In the whole-whole condition, the 6-year-olds produced more FAs than the two older groups. A separate analysis for each group showed a significant main effect of condition only for the 6-year-olds *F*(3,105) = 4.03, *p*<.01 and adults *F*(3,135) = 2.87, *p*<.05, but not for the 9–10-year-olds *F*(3,105) = 1.92, *p*>.05. The 6-year-olds produced more FAs in the whole-whole than part-part condition. The adults produced more FAs in the part-part than part-whole condition.

Taken together, these results reveal differential patterns of performance among the three age groups. Both 6- and 9–10-year-olds performed best in the part-part condition, but with a twist. The 6-year-olds’ performance showed a continuous decline as the number of whole face presentation increased (i.e., the more they saw a whole face, the worse their performance became). Although the 9–10-year-olds also followed a similar pattern, their *A′* results in the part-whole or whole-part conditions showed superior performance to those of the 6-year-olds. The adults showed a rather different pattern of *A′* results from the two groups of children in that a significant decline of performance was observed only in the whole-whole condition.

## Discussion

The main aim of the study was two fold: to examine whether children’s part recognition following part learning would be affected by the presentation of a whole face at test like adults, and to test how robust children’s part processing might be. Our results showed that like the older age groups even children in our youngest group were affected when a part of a face was shown in a whole face following part learning. This is consistent with Leder and Carbon [11] who first demonstrated this holistic interference effect in adults. However, the pattern of holistic interference in our study differed among the groups. Our results are also in agreement with numerous studies that provided evidence of children’s adult-like holistic face processing [6], [17], [18], [24]. However, our results go beyond the previous findings by demonstrating that our youngest group was most vulnerable to holistic interference than the 9–10-year-olds or adults.

In contrast to the encoding switch hypothesis that sees children’s face processing as developing from a part-based to more holistic style [4], our 6-year-olds were most affected by holistic interference. If it were true that holistic processing is only fully developed at a later age, this finding would be rather surprising because the holistic interference should have been most marked in the two older groups, rather than this youngest group. Of the three probe-test conditions, the 6-year-olds’ part recognition was worse in the part-whole or whole-part condition and worst in the whole-whole condition, relative to the part-part condition. The same pattern of results was also evident for their hit rates which showed a continuous decline as the number of a whole face in a trial increased. Moreover, they produced more FAs in the whole-whole than part-part condition. These results suggest that the presence of a whole face made part recognition difficult, regardless of whether probe and test eyes were the same or different. If the 6-year-olds’ processing were part-based, they would have shown little holistic interference.

The 9–10-year-olds also showed holistic interference, but their part recognition was less affected by the presence of a whole face. Unlike the 6-year-olds, their *A′* and hit rates did not show a continuous decline as the number of a whole face in a trial increased. In addition, there was no effect of condition on their FA rates. The adults also suffered less from holistic inference than the 6-year-olds. However, in contrast to the two groups of children, the adults’ hit rates showed holistic interference only in the whole-whole condition. On the other hand, the adults produced more FAs in the part-part than part-whole condition, and they were in general more likely to produce a ‘same’ response than the 9–10-year-olds. Such a responding bias could be linked to the increased FAs in the part-part condition.

These findings provide first evidence of holistic interference among 6- and 9–10-year-olds. In fact, the interference effect was stronger among the 6-year-olds, and this is unlikely due to their overall poorer performance as they performed as well as the two older groups in the part-part condition. Since we employed children’s faces as stimuli, the two groups of children were not disadvantaged in the task because of the stimulus choice. A more plausible interpretation is that holistic processing emerges early in development, and it may even be a default mode of face processing. Perhaps it is the ability to inhibit this default processing that develops over a longer period of life. It is possible that holistic processing is of a more automatic nature, whereas focusing on a particular feature without being strongly affected by the holistic interference requires more deliberate inhibitive efforts and experience. Our 6-year-olds were able to encode and retrieve a part as well as the two older groups, but their recognition of the part was impaired only when the part was embedded in a whole face. Therefore, our findings demonstrate that holistic processing is already present at 6 years of age, and that it is the ability to inhibit the influence of holistic information on piecemeal processing that seems to require a longer period of development into at an older and adult age.

The ability to inhibit interfering information in a scene may not be specific to face processing. Rather it may be generic information processing skills in the executive functions [15], [26]. The efficiency in response inhibition appears to develop with age, with 9-year-olds exhibiting better inhibitory processes than 7-year-olds [5]. A lack of maturity in this ability among our 6-year-olds may explain why they were most vulnerable to holistic interference.

Inhibitory processes may be particularly useful when a face needs to be recognised after certain facial features have gone through some changes. For instance, an eye suffering from an infection or physical trauma often results in a change of the shape and colour (e.g., swollen or blackened). However, in order to still recognise this face as the same person, it is important to inhibit the influence of this altered feature. Under the influence of holistic processing, a change to such a key facial feature can affect the perceived shape of the whole face. This gestalt perception could result in misidentification. Thus an inhibitory control of holistic processing can be beneficial in this kind of situations, and that younger children may be less capable to identify a face in similar situations. Moreover, inhibitory mechanisms may also be important under a forensic setting whereby the police ask an eyewitness to construct the perpetrator’s face using a face composite system. Early systems (e.g., Identikit or Photo-Fit) rely on a feature based construction which could be disadvantageous to younger children who lack in inhibitory skills because they would be less able to focus on an individual facial feature while inhibiting the influence from other facial features.

Although our main findings are fairly clear-cut, some of the details are less so. For example, it is not clear why the adults were not affected by the part-whole and whole-part conditions relative to the part-part condition and why they and the 9–10-year olds were not equally affected by the part-whole/whole-part and whole-whole conditions. It could be that the adults were better able to separate the eye region from the rest of face than the 9–10-year-olds. This could have enabled them to form a clearer representation of the part, which may have minimised the effects of different conditions. The ability to create a visual imagery of a segmented eye region may rely on the maturity of visuo-spatial working memory, which is known to have a limited capacity [1], [2], with a gradual development throughout childhood and early adulthood [10], [20]. Adults should have a fully developed capacity hence can store more elaborate visual information than children.

In the part-whole condition, a part had to be extracted from a whole face in the visual working memory at test in order to compare the visual representation of the extracted part with that of the part during encoding. Similar processing needed to take place in the whole-part condition whereby a part had to be segmented from a whole face during encoding in order to decide whether the visual representation of the extracted part resembled the part at test. This means that the whole-whole condition should have been most demanding as it required the segmentation of the eye region twice in the visuo-spatial working memory, whereas the part-whole or whole-part condition only required either segmentation or representation of a critical part, but not both.

We compared the part-whole and whole-part conditions to identify whether the locus of the holistic interference can be attributed to the stage of encoding or retrieval. Since none of our age groups showed a difference in performance between these conditions, it is likely that the probe-test matching process alone was responsible for the holistic interference effect in all age groups.

Our findings demonstrate that the ability to tackle the challenging processing was different among different age groups. A less developed visual working memory may explain why children in our study were less able to separate and form a clear representation of a facial part in comparison to the adults. Leder and Carbon [11] suggest that the difficulty to ignore irrelevant parts in a face may reflect the essence of holistic face processing. Our results demonstrate that this holistic interference is not only already present at a young age, but is also stronger at this age than at an older or adult age.
